# The association between gender expression, beliefs about alcohol, coping skills, and alcohol consumption in post-secondary students at two Canadian universities

**DOI:** 10.1192/j.eurpsy.2022.625

**Published:** 2022-09-01

**Authors:** A. Bahji

**Affiliations:** University of Calgary, Department Of Psychiatry, Calgary, Canada

**Keywords:** Survey, University Mental Health, gender, alcohol

## Abstract

**Introduction:**

Unlike sex, the association between gender and high-risk drinking has been relatively understudied in post-secondary students. Gender expression may influence the use of protective coping strategies and beliefs about alcohol.

**Objectives:**

This study evaluated associations between gender expression, protective coping strategies, beliefs about alcohol, and high-risk alcohol use in post-secondary students.

**Methods:**

We analyzed data from a cross-sectional study of 3,446 undergraduate students at two Canadian universities in October 2017. The primary outcome was high-risk drinking during the previous month, measured by the Alcohol Use Disorders Identification Test (AUDIT) score. We evaluated gender expression (masculine, feminine, androgynous, and undifferentiated), protective coping strategies, and beliefs about alcohol using validated scales. Multivariable logistic regression models were used to test the association between gender expression and AUDIT scores.

**Results:**

The most prevalent gender expression was androgynous (35.1% overall), while the undifferentiated role was the least prevalent (17.4% overall). Those who adhered to an androgynous gender role (OR = 1.45, 95% CI: 1.10, 1.90) were significantly more likely to engage in problem drinking. In addition, greater scores on the protective behavioural strategies scale were associated with reduced odds of problem drinking (OR = 0.96; 95% CI: 0.95, 0.97) while higher alcohol saliency scores were associated with higher odds of problem drinking (OR = 1.12; 95% CI: 1.10, 1.13).

**Conclusions:**

Higher protective behavioural strategies and lower alcohol salience beliefs were associated with lower alcohol use. Androgynous gender roles were associated with high-risk alcohol use.

**Disclosure:**

No significant relationships.

